# The correlation between dengue incidence and diurnal ranges of temperature of Colombo district, Sri Lanka 2005–2014

**DOI:** 10.3402/gha.v9.32267

**Published:** 2016-08-25

**Authors:** N. D. B. Ehelepola, Kusalika Ariyaratne

**Affiliations:** 1 The Teaching (General) Hospital – Kandy, Kandy, Sri Lanka; 2 Lanka Hydraulic Institute, Moratuwa, Sri Lanka

**Keywords:** dengue, diurnal temperature range, mathematical models, climate change, urban heat islands

## Abstract

**Background:**

Meteorological factors affect dengue transmission. Mechanisms of the way in which different diurnal temperatures, ranging around different mean temperatures, influence dengue transmission were published after 2011.

**Objective:**

We endeavored to determine the correlation between dengue incidence and diurnal temperature ranges (DTRs) in Colombo district, Sri Lanka, and to explore the possibilities of using our findings to improve control of dengue.

**Design:**

We calculated the weekly dengue incidence in Colombo during 2005–2014, after data on all of the reported dengue patients and estimated mid-year populations were collected. We obtained daily maximum and minimum temperatures from two Colombo weather stations, averaged, and converted them into weekly data. Weekly averages of DTR versus dengue incidence graphs were plotted and correlations observed. The count of days per week with a DTR of >7.5°C and <7.5°C were also calculated. Wavelet time series analysis was performed to determine the correlation between dengue incidence and DTR.

**Results:**

We obtained a negative correlation between dengue incidence and a DTR>7.5°C with an 8-week lag period, and a positive correlation between dengue incidence and a DTR<7.5°C, also with an 8-week lag.

**Conclusions:**

Large DTRs were negatively correlated with dengue transmission in Colombo district. We propose to take advantage of that in local dengue control efforts. Our results agree with previous studies on the topic and with a mathematical model of relative vectorial capacity of *Aedes aegypti*. Global warming and declining DTR are likely to favor a rise of dengue, and we suggest a simple method to mitigate this.

## Introduction

Dengue is a viral infection with life-threatening forms, transmitted by *Aedes* mosquitoes, and a major global public health problem (1). Meteorological factors, such as rainfall, count of rainy (and wet) days, humidity, temperature, wind, and duration of sunshine, have been demonstrated to influence dengue incidence in hundreds of studies performed around the world (2–4). Even though most of them have studied the relationship between dengue transmission and temperature, only very few have analyzed the correlation between dengue and diurnal ranges of temperature (4). Other factors, such as herd immunity, introduction of new virus phenotypes to the population, efficiency of preventive measures, population movements, urbanization, housing and refuse disposal methods, and knowledge and attitudes of the public, are also known to influence dengue incidence.

Diurnal temperature range (DTR) is the difference between daily maximum and minimum temperatures. Several studies concerning DTR dengue correlation were carried out after the first publication appeared in 2011 (4). We recently demonstrated that dengue incidence in Kandy city, in the central hill country of Sri Lanka, is correlated with DTR (5).

We decided to perform this study for the following reasons: Dengue is now endemic in various localities in more than 100 countries (1), hyper endemic in Sri Lanka, and one of the world's fastest spreading infections. Although dengue is a global problem, its correlation with DTR has so far only been epidemiologically demonstrated in three localities: Thailand, Bangladesh, and Sri Lanka (4–7). There is a local (Sri Lanka) and global tendency of declining DTR with ongoing climate change (8, 9), which may further facilitate dengue transmission in many areas around the globe (6). Life traits of *Aedes aegypti* species from different geographical locations were shown to be varied by some laboratory studies (7). Considering the previously mentioned reasons, the need for more epidemiological studies on the topic in more localities is clear. Entomological studies carried out in laboratories mimicking DTR in Mae Sot, Thailand, have shown mechanisms of how DTR affects dengue transmission (4, 7, 10). *Aedes* vectors’ lifespans are shorter, and their susceptibility to dengue virus infection is reduced, when DTRs fluctuate widely around the same mean temperature, when this temperature is >18°C, as in Colombo (4). Large DTRs prolong the aquatic part of the *Aedes* life cycle, lower larval survival rates, and reduce adult female reproductive output (7). Wide fluctuations of DTR around a mean of 26°C reduce midgut infection rates of the vector and tend to increase the dengue virus's extrinsic incubation period (10). It is important to ascertain the applicability of those laboratory findings to the real world, through epidemiological studies.

**Fig. 1:**
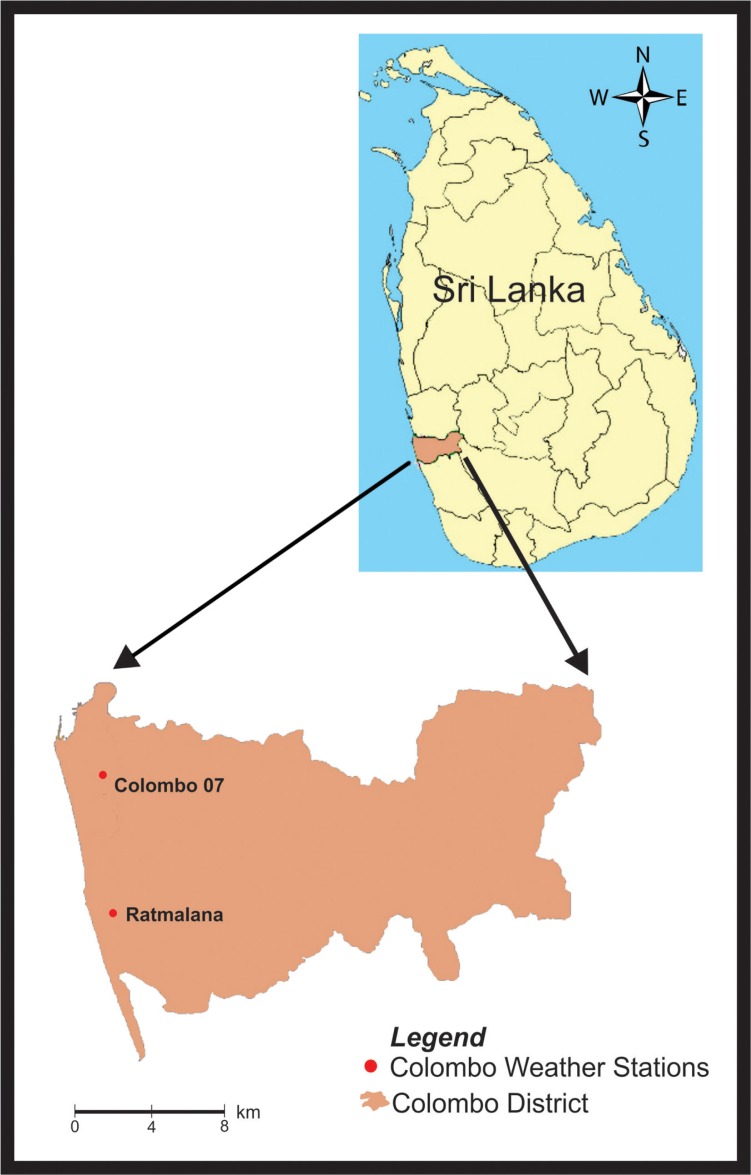
A map of Sri Lanka (inset) and our study area. Colombo district in the west coast is shaded in light brown color in contrast to the other 24 districts. Locations of the two weather stations from which we obtained our weather data are depicted.

Previous locations where dengue DTR correlation was studied (Mae Sot, Dhaka, Kandy) have an approximate mean annual temperature of 25°C–26°C and annual rainfall below 2,000 mm. In contrast, we estimated for our study period that Colombo district had a mean temperature of 28.1°C (average of two weather stations), with annual rainfall in Colombo being approximately 2,350 mm (11). Colombo is closer to the equator and has a lower average DTR (typically DTR becomes smaller toward the equator) compared with the above three locations. Colombo district, located in the western coast low lands of Sri Lanka (Fig. 1), has a warmer and wetter climate than Kandy (located in the central highlands) and has a lower DTR (DTR typically increases with elevation).

We have estimated two dengue incidences of Sri Lanka. In 2005, it was 31 per 100,000 population, but increased to 228 per 100,000 population in 2014 (12, 13). We used population data from the Sri Lanka Department of Census and Statistics for this estimation. However, those reported dengue cases represent only a fraction of total cases (2). By comparison, one estimate shows the average dengue incidence of the World Health Organization's (WHO) Southeast Asia region for the 2000–2010 period as 13 per 100,000 population (14). This is the world's most severely affected region by dengue and includes Sri Lanka. Therefore, the burden of dengue in Sri Lanka is clearly greater than the average for this region. The dengue case fatality rate in Sri Lanka was 0.5% in 2005, increasing to 1% in 2009 and falling to 0.2% in 2014 (12, 13).

There is an interesting mathematical model regarding changes of relative vectorial capacity (rVc) of *Aedes aegypti* mosquitoes in different DTRs around varying mean temperatures (15). By performing several epidemiological studies, we can ascertain the usefulness of that model in the real world and further improve it. Such models can be used to forecast future dengue incidences with ongoing changes of DTR due to climate change. Reliable predictions will allow better preparation and therefore better management of dengue.

### Study setting

Colombo has a tropical wet climate, category Af according to the *Koppen Geiger classification* (11). We estimated the mean daily temperature of Colombo district as 28.1°C, and the minimum and maximum average temperatures for our study period as 24.9°C and 31.2°C, respectively.

Colombo's population, according to the 2012 census, was 2.31 million. Colombo is the smallest (699 km^2^), but most densely populated (3,333 people per km^2^), district in Sri Lanka.

### Objectives and hypotheses

Our objectives were to determine the correlation between Colombo's dengue incidence and DTR, and compare our results with those of similar studies. Then, we endeavored to explore possible ways to improve dengue control using those correlation patterns, as well as other available evidence. Our hypothesis was that a large DTR has a negative correlation with the incidence of dengue in Colombo.

## Method

### Ethical statement

Only notified dengue patient numbers were collected (without any information about their identity). We obtained clearance (exemption from detailed ethics review) from the ethical review committee of the Peradeniya medical faculty (2015/EC/25).

### Data

The numbers of notified dengue cases from the Colombo district from January 1, 2005 to December 31, 2014 were collected by analyzing weekly health ministry epidemiology reports. Mid-year population data of Colombo for the same period were obtained from the Department of Census and Statistics. Daily minimum and maximum temperatures of both weather stations of the Sri Lanka Department of Meteorology in Colombo district (situated at Colombo seven and Ratmalana=Rathmalana) relevant to our study period were purchased. We decided to take average values of both weather stations, in order to compensate for differences in weather within the district, as there were many people who live in one part and work in another part of Colombo. Most *Aedes* mosquitoes spend their entire life near the dwelling where they were born (16); so data of a distant weather station may be of little relevance to their life cycle. Hence, we selected weather stations within the study area.

### Analysis

The weekly dengue incidence of Colombo for 2005–2014 was calculated. Temperature data were converted to weekly values and averaged. Weekly averages of diurnal ranges of temperature were also calculated.

**Fig. 2:**
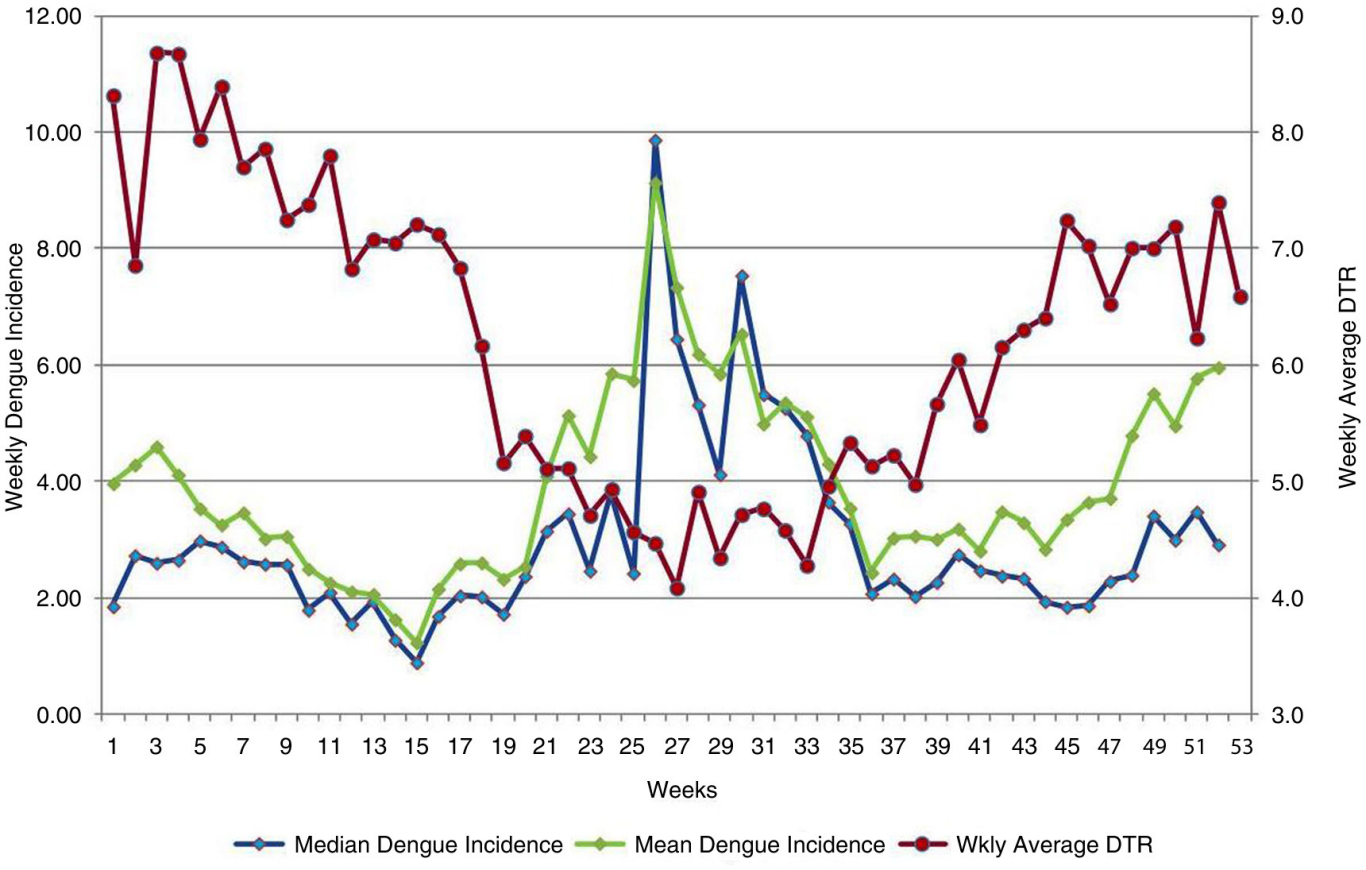
Changes in weekly average diurnal temperature range (DTR) in °C (red), and weekly dengue incidence over the course of 52 weeks of the year, 2005–2014. *x*-axis: weeks; primary *y*-axis: median weekly dengue incidence (blue) and mean weekly dengue incidence (green); secondary *y*-axis: DTR in °C.

**Fig. 3:**
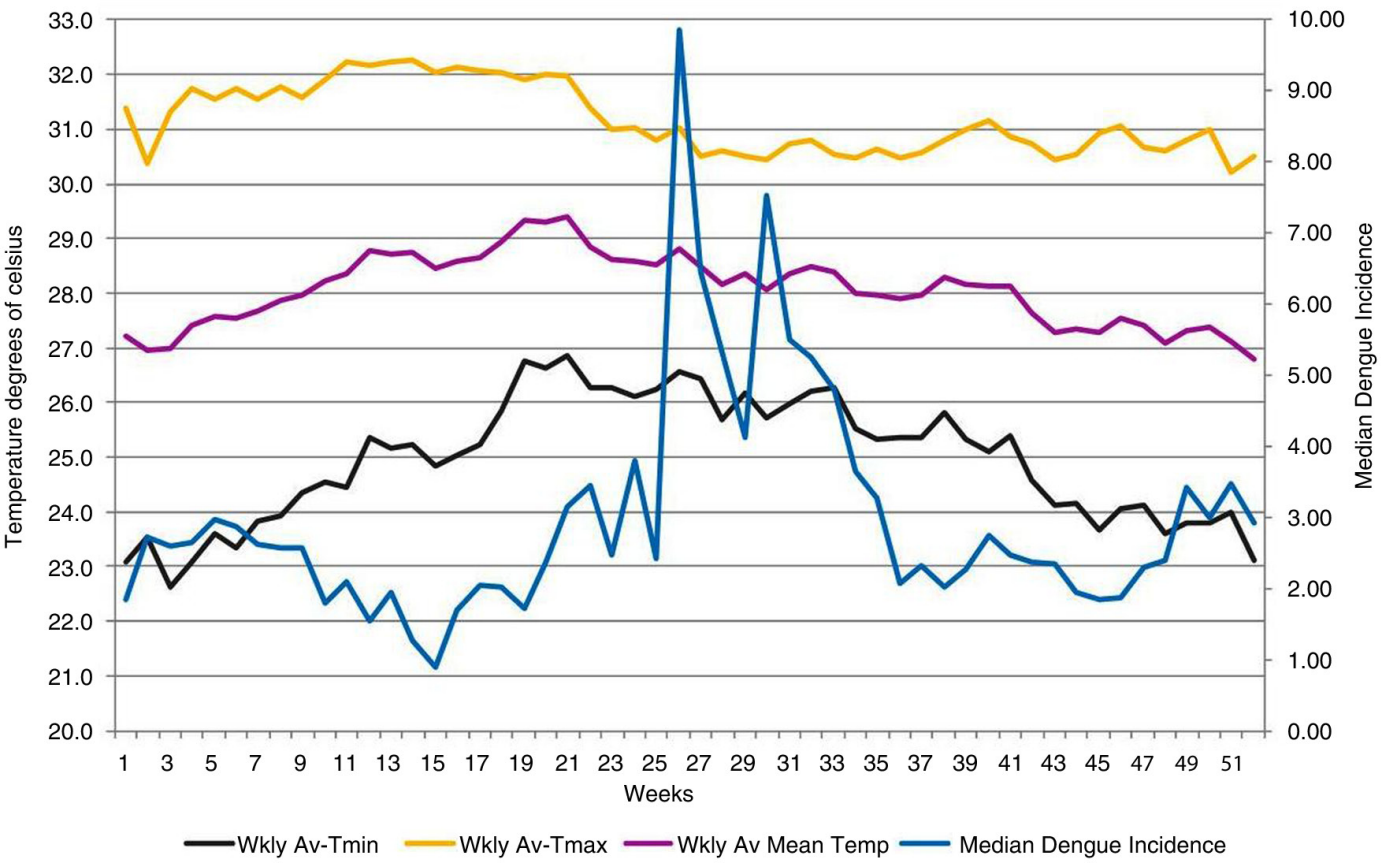
Changes in weekly average minimum (black), maximum (yellow), and mean (purple) temperatures and median weekly dengue incidence over the course of all 52 weeks of the year, for 2005–2014. *x*-axis: weeks; primary *y*-axis: temperature in °C; secondary *y*-axis: median weekly dengue incidence (blue).

We plotted time series graphs of median and mean weekly dengue incidences versus weekly averages of DTRs during the course of 52 weeks of the year and observed correlation patterns (Figs. 2 and 3).

We observed that a DTR>7.5°C is followed by a decline of dengue incidence after several weeks (however, a DTR>7.5°C is not intended to be a precise threshold value). We wanted to further verify the observed pattern by analyzing data in another way, using the wavelet analysis technique. During a particular week, there are sometimes several days with extreme DTR values. We performed wavelet analyses to determine the relationship between the count of days per week where DTR was >7.5°C and <7.5°C versus the weekly dengue incidence.

Wavelet analysis was carried out following the same procedure as per our Kandy study, published in this journal (5). Here we give a brief description; cross wavelet transform (XWT) and wavelet coherence (WTC) can be used for examining relationships in time frequency space between two time series. A large common power in XWT and a consistent phase relationship in WTC indicate causality between the two time series (17).

Continuous wavelet transform (CWT) was calculated for count of days per week with a DTR>7.5°C and <7.5°C. Then, XWT and WTC were calculated for each of them and dengue incidence. Vectors were indicative of the phase difference. A horizontal arrow pointing from left to right signifies ‘in phase’, with an arrow pointing vertically upward indicating the second series lagging the first by 90°. WTC is defined as the square of the cross-spectrum, normalized by the individual power spectra. This gives a quantity between 1 and 0, and measures the cross-correlation between two time series, as a function of frequency.

Because the wavelet transform is a band-pass filter with a known wavelet function, by summing over a subset of the scales it is possible to reconstruct the original time series. In this study, the period, which gave the highest coherence among the dengue incidences, and each of the DTR variables were identified. The wavelet filtered time series for this period was reconstructed, and the lagging time was estimated.

MATLAB R2013a software was used for wavelet analysis, and Microsoft Office 2007 software was used for other work.

## Results

Figures 2 and 3 clearly illustrate that large weekly DTRs were followed by a reduction of dengue incidence after several weeks.

According to Fig. 3, from weeks 22 to 37 the mean weekly temperature was between 28 and 29°C; from Fig. 2, it can be seen that between weeks 23 and 34, DTR was lowest (4°C–5°C). Both mean and median dengue incidence values increased after a few weeks lag. According to Figs. 2 and 3, from weeks 3 to 9 the mean temperature was lower (27°C–28°C) and the DTR was higher (7.2°C–8.7°C). DTR was highest during the fourth week. We observed a fall in both median and mean dengue incidences after a lag of several weeks (Figs. 1 and 3).

**Fig. 4:**
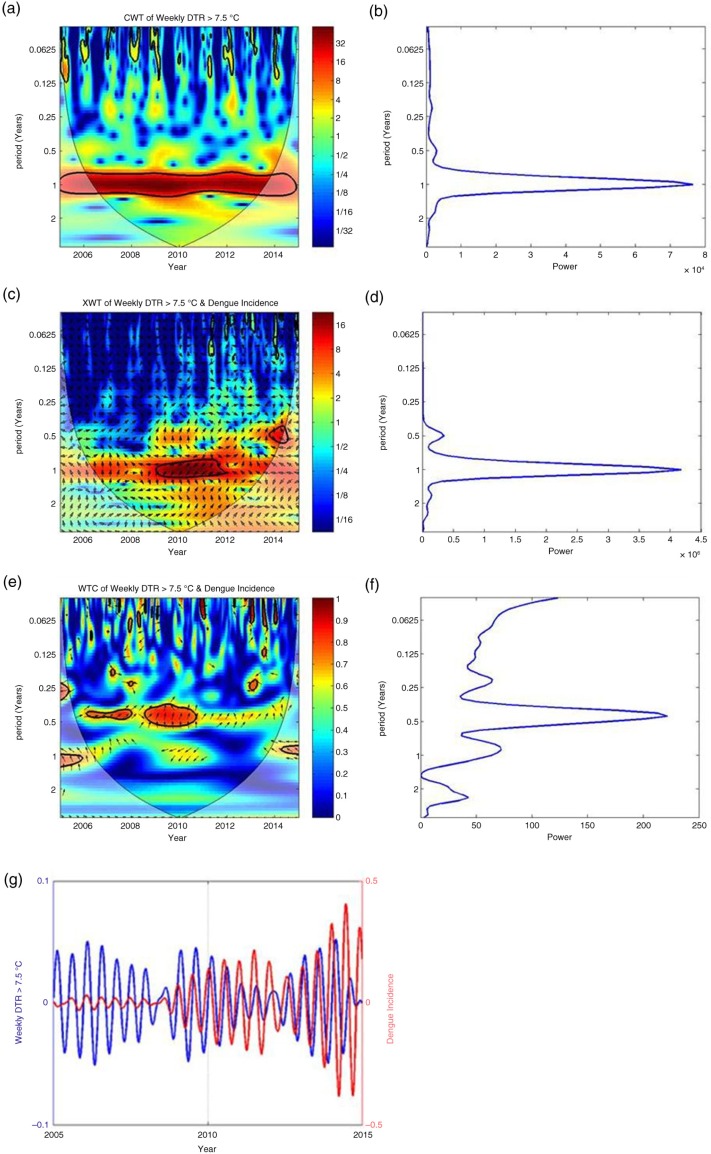
Results of wavelet analyses of weekly dengue incidence versus the count of days per week with a DTR>7.5°C: (a) continuous wavelet transform (CWT) variations; (b) wavelet power of CWT; (c) crosswavelet transform (XWT) variations; (d) wavelet power of XWT; (e) wavelet coherence (WTC); (f) wavelet power of WTC; and (g) reconstructed time series for selected periods. The term ‘period’ in the vertical axis indicates duration of cycle (in years). For a, c, and e, there are color-coded columns on the right side of the main figure. They indicate the strength of coherence, in which dark blue and dark red indicate lowest and highest coherence, respectively.

Wavelet analyses showed that the count of days with a DTR>7.5°C per week and weekly dengue incidence were negatively correlated, with an 8 (7.9) week lag. The count of days with a DTR<7.5°C per week and weekly dengue incidence were positively correlated, with an 8 (7.8) week lag. Figure 4 illustrates the results of the wavelet analysis of weekly dengue incidence versus the count of days per week with a DTR>7.5°C.

## Discussion

The study's results confirmed our hypothesis of large DTRs having negative correlations with dengue incidence in Colombo. This was shown by our time series graphs and further confirmed by results of our wavelet analyses. We also observed that small DTRs were favorable for dengue transmission. The correlation patterns we found were similar to those from studies carried out in Kandy, Sri Lanka, for 2003–2012 (5), and in Dhaka, Bangladesh, for 2000–2009 (6). However, the lag periods in our study are longer. No information is available regarding lag periods in Mae Sot, Thailand. Past dengue incidence versus meteorological factors correlation studies have shown that lag periods vary from a few weeks to a few months, in various localities (2). Hence, our result is not unusual, although we could not determine the precise reason for longer lag periods. In Sri Lanka, once a vector with dengue virus in its saliva bites a healthy person, it usually takes 2–3 weeks for that person to develop symptoms, go to a hospital, get diagnosed, and then get reported (2).

**Fig. 5:**
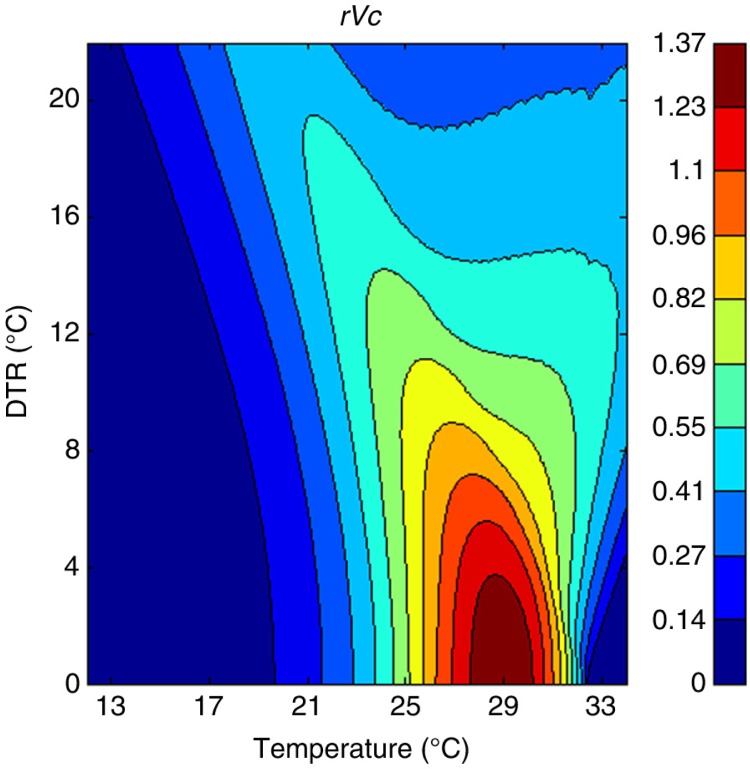
The effect of temperature and DTR on the relative vectorial capacity (rVc) of *Aedes aegypti* mosquitoes. The color-coded column on the right side of the graph describes the rVc value. A higher rVc corresponds to increased dengue epidemic potential.

Graphs depicting rVc of the *Aedes* vector according to different DTRs around different mean temperatures already exist, such as Fig. 1 of reference (15). We use a part of that here as Fig. 5.

This graph was developed by theoretical mathematical modeling of dengue transmission. As described previously, in Fig. 3 from weeks 22 to 37 the mean weekly temperature was between 28 and 29°C, and according to Fig. 2, between weeks 23 and 34 DTR was lowest (4°C–5°C). According to the rVc graph (Fig. 5), this combination of mean temperature and DTR enhances rVc of the vector mosquito. Therefore, it is highly conducive for dengue transmission. This is confirmed in Fig. 2 by the increase of mean and median dengue incidence values following a lag period. According to Figs. 2 and 3, from weeks 3 to 9 the mean temperature was lower (27°C–28°C) and DTR was higher (7.2°C–8.7°C). DTR was highest during the fourth week. We observe a decrease of both median and mean dengue incidences after several weeks (Figs. 2 and 3). These findings are also in agreement with the rVc graph. Both *Ae. aegypti* and *Aedes albopictus* vectors are present in Colombo district, but information regarding their ratios of contribution to Colombo dengue incidence is unavailable at present. There are differences in their biology, and it is interesting to see this theoretical model (15) (which considers only *Ae. aegypti* as dengue vector) agrees with our findings, which were derived from epidemiological data in a place where two dengue vectors are found. We found a similar occurrence in our Kandy study as well (5).

In Kandy, with an average mean temperature of 25.1°C, when DTR is >10°C, dengue incidence declines after a lag period (5). In Colombo, with an average mean temperature of 28.1°C, a lower DTR (>7.5°C) causes similar phenomena. This also agrees with the rVc graph (Fig. 5). Results of the present and Kandy study (5) illustrate that an 8.5°C DTR around mean temperatures 25.1°C and 28.1°C results in opposite effects on dengue transmission in Kandy and Colombo, respectively. We propose a possible explanation of this, in the context of the rVc of the vector, as follows: In the rVc graph, if we consider a zero DTR as the baseline, an 8.5°C DTR reduces the rVc more steeply when mean temperature is 28.1°C, compared with a mean of 25.1°C. Therefore, it appears that dengue transmission in Kandy and Colombo generally follows the pattern of rVc of the vector. This supports the validity, and therefore the usefulness, of this mathematical model in the real world. However, we have also noted the following weakness. In Colombo during week 5, there was a DTR of 7.9°C around a mean temperature of 27.6°C (Fig. 3), which is unfavorable for dengue transmission. Nonetheless, that combination corresponds to a larger rVc than when the mean temperature was 25°C–25.5°C, even with a zero DTR (Fig. 5). However, in Kandy a DTR of 6.5°C–7°C around a 25°C–25.5°C mean was also highly conducive for dengue transmission, as depicted in Fig. 2 of reference (5). Here, our findings disagree with the rVc graph.

**Fig. 6:**
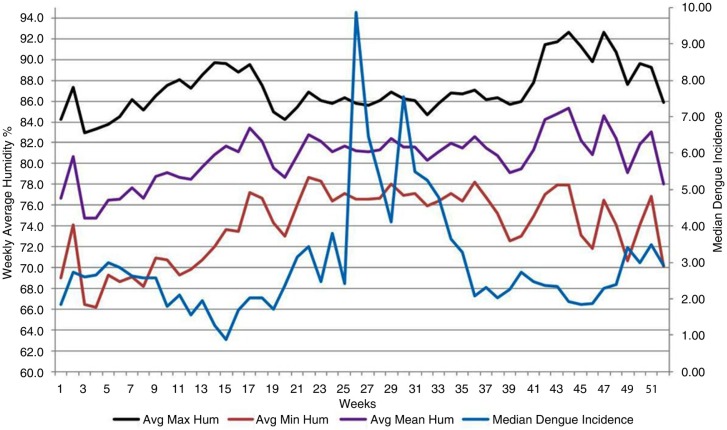
Changes in weekly average minimum (brown), maximum (black), and mean (purple) humidity% and median weekly dengue incidence, over the course of all 52 weeks of the year, for 2005–2014. *x*-axis: weeks; primary *y*-axis: weekly average humidity; secondary *y*-axis: median weekly dengue incidence (blue).

Our Fig. 2 illustrates a small rise of dengue incidence in the 38–52 weeks, despite a rising DTR during this period. The possible explanations are as follows. One likely reason is that during this period, Colombo receives more rain compared with the first weeks of the year (Colombo receives its lowest rainfall in January and February). Rainfall has also been demonstrated to be correlated with dengue incidence in Colombo (18). We suggest this may be due to another reason. Higher humidity at the same temperature has been demonstrated to increase the longevity of the *Aedes* vector (19). We made a time series graph of humidity for our study period, using data from the same two weather stations, in order to explore this possibility. Accordingly (Fig. 6), average humidity remained higher (>80%) during the 38–52 weeks, compared with the first 13 weeks (75–80% range).

Mean temperatures remained similar during weeks 38–51 and the first 13 weeks (Fig. 3). Increase of longevity exponentially increases rVc of the vector (4). However, Colombo's mean temperatures remained around 28°C during this period, whilst that entomological study was performed at 25°C.

During the first 13 weeks, the magnitude of DTR was higher in Colombo (Fig. 2), and average humidity was lower (Fig. 6). We propose that the lowest dengue incidence, seen in week 15, can be attributed to a high DTR of preceding weeks. Low rainfall and average humidity may also have contributed to this to a certain extent.

### Possible ways to use information gathered for improvement of dengue control

In all places where local dengue DTR correlation has been studied in detail so far (Mae Sot, Dhaka, Kandy, and Colombo), mean temperature was relatively low (but in the 18°C–33°C range), and DTR was higher during the first quarter of the year, and was unfavorable for dengue transmission. This period corresponds to the winter in the Northern Hemisphere. On a global scale, winter warming due to global climate changes is more rapid than summer warming (9, 20). Increases of minimum temperatures are greater than the rise of maximum temperatures in Sri Lanka and in the world, although both of them rise (8, 9). However, this does not happen everywhere on the earth. This implies that in many places in the Northern Hemisphere during the first quarter of the year, the mean temperature is likely to rise, and DTR is likely to become smaller in the coming decades. When we consider the effect of these changes on the life cycles of the *Aedes* vector and the dengue virus (as discussed in the introduction), it is likely to result in more vectors with dengue virus in their saliva, and they will bite more frequently with rising temperatures (5). Considering the great majority of the world's population, and more specifically the population at risk of getting dengue, live in the Northern hemisphere, we may expect more dengue patients even during the first quarter of the year, with ongoing climate changes. To our best knowledge, there have been no previous publications regarding this risk. We also believe that there is a simple, sustainable, and feasible method to mitigate this problem. We propose to popularize local application of mosquito repellents, particularly in the dawn and evenings. This will supplement existing dengue control programs in Colombo and in other areas with high risk of dengue, especially during periods with low DTR and high average temperatures. This proposal has many additional benefits (2, 5).

In Colombo and other parts of Sri Lanka, the cornerstone of dengue prevention is elimination of vector breeding sites and immature forms of the vector. These preventive activities become vigorous at the onset of the monsoon rain seasons and when dengue incidence rises. Even though the smallest in area, Colombo is the hub of the nation's administration and economy and makes the largest contribution to the gross national product. At the same time, Colombo district also typically reports the highest number of dengue cases. Hence, control of dengue in Colombo is very important. Despite vigorous preventive campaigns, Colombo's dengue incidence was 81 per 100,000 population in 2005 and rose to 622 per 100,000 population in 2014.

Therefore, there is a clear need for additional (or alternative) efforts to control dengue in Colombo. We suggest maintaining vigorous dengue control programs during the first quarter of the year, and capitulate on the weather conditions not conducive for dengue transmission (shown in Figs. 2 and 3), and maximally suppress dengue transmission during this period. This will help minimize the chances of an epidemic during subsequent weeks.

In the recent past, there have been many advances in the use of sophisticated mathematical models in dengue epidemiology and in other areas of public health. This is a relatively new, little known concept to most Sri Lankan (and most other developing world) health care workers. We hope our findings of the validity and potential uses of a mathematical model of rVc of the dengue vector (15) will generate interest among our colleagues regarding potential uses of these tools in common public health problems.

### Limitations of the study

Reliability of our results depends upon the fidelity of our data. Notified dengue cases are only the tip of the dengue iceberg. A study performed in Colombo in 2008 showed the existence of approximately 30 primary dengue cases in children (<12 years) for every single case that was notified (21). However, notified dengue cases were the best practical option available to us.

Urban heat islands (UHI) and cold islands have been demonstrated in Colombo district (22, 23). In one study (23), a difference in temperature up to 7°C was found between different urban locations, and marked temperature differences between sunlit and shaded areas were recorded during the daytime. Nocturnal heat islands, which were 3°C warmer than the surroundings, were also found. The pattern of change of both temperature and humidity during the course of a day differs in UHIs (22, 23). There are two weather stations for the Department of Meteorology in the district, and we took averages of the recordings of both of them, in order to compensate for intradistrict differences in weather. Most of the heat islands and the majority of the population are on the western side of the district, and both of our weather stations are also from the western side of the district. UHIs in Colombo (and elsewhere) are typically areas with high population density and large floating populations. There, the temperatures are higher in mornings and evenings (23). The *Aedes* vector bites more frequently in the mornings and evenings, and higher temperatures further increase biting rates. This is more conducive for dengue transmission. However, considering the rVc graph (Fig. 5), the mean temperatures of >31°C are not particularly favorable for dengue transmission. DTRs are also higher in UHIs, which are unfavorable for dengue transmission, but large DTRs around a mean of >33°C again favor dengue transmission. Dedicated future studies may help us to better understand dengue dynamics in UHIs. There are hundreds of published studies on dengue temperature correlation (2) in urban areas. To our best knowledge, none of the other researchers considered the possible influence of UHIs on their results.

## Conclusions

Large DTRs were negatively correlated with dengue transmission in Colombo district. An existing mathematical model of rVc of *Ae. aegypti* in relation to different average temperatures, and different DTRs around them, agrees with our findings. Such models are likely to be useful for studying and predicting changes in dengue epidemiology due to climate change. Our detection of a negative correlation between large DTR and dengue incidence in Kandy was confirmed in Colombo. Similar studies in areas with different climate and dengue transmission patterns will help to further verify our findings.

## Paper context

Entomological studies and a mathematical model show diurnal temperature range (DTR) affects dengue transmission, but there is a paucity of epidemiological evidence. We have demonstrated that large DTRs are unfavorable for dengue transmission in Colombo district, Sri Lanka. We propose ways to use our findings to improve local dengue control and to mitigate a potential rise of dengue due to ongoing global declines in DTR.
